# Chondrosarcoma of Ureter in an Elderly Patient: A Case Report

**DOI:** 10.3390/medicina59030454

**Published:** 2023-02-24

**Authors:** Szu-Ying Pan, Chien-Zhi Lai, Wen-Chi Chen, Yung-Hsiang Chen, Che-Hung Lin, Han Chang, Chi-Ping Huang, Ching-Chung Yeh

**Affiliations:** 1 Department of Urology, China Medical University Hospital, Taichung 404332, Taiwan; 2 School of Medicine, College of Medicine, China Medical University, Taichung 404333, Taiwan; 3 Graduate Institute of Integrated Medicine, College of Chinese Medicine, China Medical University, Taichung 404333, Taiwan; 4 Department of Psychology, College of Medical and Health Science, Asia University, Taichung 413305, Taiwan; 5 Division of Oncology, Department of Internal Medicine, China Medical University Hospital, Taichung 404332, Taiwan; 6 Department of Pathology, China Medical University Hospital, Taichung 404332, Taiwan

**Keywords:** chondrosarcoma, ureter, fluorouracil, nephroureterectomy, stone

## Abstract

Chondrosarcoma is a rare type of cancer that can affect the upper urinary tract. Because of its rarity, the clinical presentation of chondrosarcoma can be similar to other urinary tract conditions, such as renal colic, hematuria, and urothelial carcinoma. The primary treatment for chondrosarcoma is the surgical removal of the tumor, and radiation or chemotherapy may be used for advanced cases. However, because of the limited number of patients with this condition, there are no established guidelines for chemotherapy, and the outcomes are unclear. In this case, we present a 71-year-old female patient who was diagnosed with ureteral chondrosarcoma. She presented with abdominal pain and hydronephrosis, and a tumor was found beneath a small stone. The patient underwent nephroureterectomy and received oral fluorouracil chemotherapy due to the advanced stage of the disease. Fortunately, the patient survived, and at the 7 months post-operative follow-up there was no evidence of recurrence. In conclusion, the chondrosarcoma of the upper urinary tract is a rare condition that can be difficult to diagnose due to its similarity to other urinary tract conditions. Treatment typically involves the surgical removal of the tumor, with radiation or chemotherapy reserved for advanced cases. However, because of the limited number of patients, there are no established guidelines for chemotherapy, and the outcomes of treatment are unclear.

## 1. Introduction

Chondrosarcoma is a frequently seen bone neoplasm, accounting for approximately 20% of all bone tumors. It is a malignant tumor that arises from chondrocytes, which are the cells that produce cartilage [1,2]. Most extra-skeletal chondrosarcomas occur at the limbs, with only 10~13% of primary chondrosarcomas occurring in the retroperitoneum, abdomen, and pelvis [3,4]. Primary renal or ureteral chondrosarcoma has rarely been reported, and is considered a very rare entity [5,6,7,8]. The youngest patients with reported cases of renal or ureteral chondrosarcoma were a 22-year-old female Indian patient and a 27-year-old white male. All other cases were aged over 60 years, with the average age of onset being in the seventh decade of life [6,8,9].

Röckelein et al. reported a case of chondrosarcoma presenting with acute colicky pain and hydronephrosis [7]. The patient received surgical treatment with nephroureterectomy; however, the clinical outcome was poor and the patient died 2 months after the diagnosis. This highlights the poor prognosis associated with chondrosarcoma of the ureter. Xu et al. reported a patient with left kidney chondrosarcoma with ureteral infiltrating urothelial carcinoma [8]. They performed multitarget fluorescence in situ hybridization (FISH) and concluded that the development of the urothelial carcinoma was triggered or induced by the chondrosarcoma component. This raises the question of whether chondrosarcoma could potentially play a role in the development of other tumors, and further research is needed to clarify the pathogenesis of the chondrosarcoma of the ureter, given its rarity in clinical practice. 

Although chondrosarcoma is a well-known bone tumor, primary renal or ureteral chondrosarcoma is a rare entity, and the prognosis is generally poor. Clinicians should be aware of the possibility of chondrosarcoma in the differential diagnosis of ureteral tumors, and further research is needed to better understand the pathogenesis and treatment of this rare tumor.

The clinical presentation of urinary chondrosarcoma includes abdominal pain, renal colic, and hematuria, but it may also be asymptomatic and found incidentally [10]. We report a female patient with ureteral chondrosarcoma in whom the clinical presentation mimicked stone disease with obstructive uropathy. We also report the outcome after surgery and chemotherapy.

## 2. Case Report

**Figure 1:**
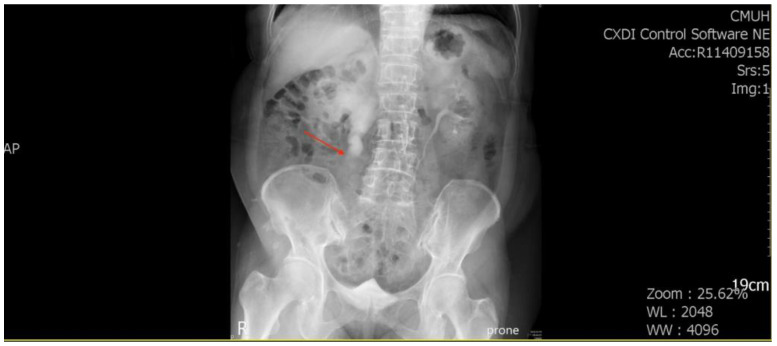
Intravenous pyelography revealed an obstruction at the right upper ureter (arrowhead).

A 71-year-old female patient with a history of recurrent stone disease presented with abdominal pain. She denied a history of diabetes and hypertension. She had been treated for bilateral urolithiasis for 12 years, including a left kidney stone that had been treated with extracorporeal shock wave lithotripsy (ESWL), a right ureteral stone that had been treated with ureterorenoscopic surgery, and a right renal stone that had been treated with ESWL. She presented with symptoms of painless gross hematuria and abdominal pain in May 2022. The renal echo revealed right hydronephrosis and a small renal stone. Intravenous pyelography showed a small upper ureteral stone with obstruction at the right upper ureter (Figure 1). 

**Figure 2:**
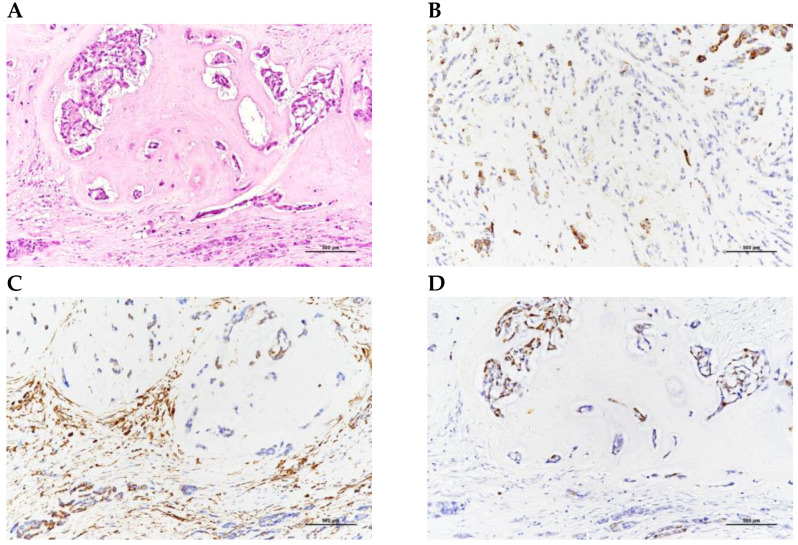
Pathological sections with immunoprofile. (**A**) HE stain; (**B**) CK (+); (**C**) vimentin (+); and (**D**) S100 (+).

**Figure 3:**
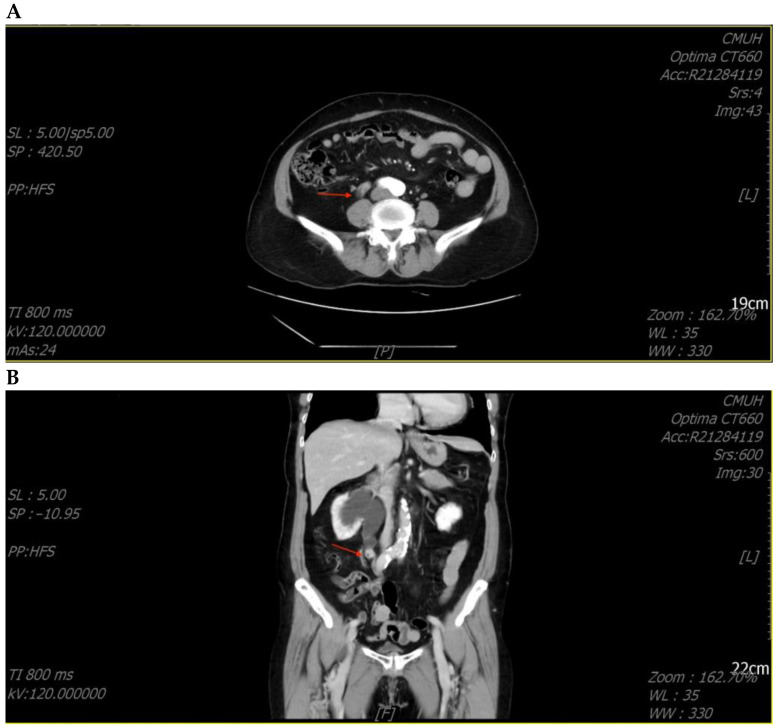
Computed tomography of the abdomen revealed a tumor (arrowhead) in the right upper ureter with proximal dilatation. (**A**) Coronary view; (**B**) Sagittal view.

She underwent ureterorenoscopic surgery under the impression of a ureteral stone with obstruction. During the operation, a small stone was found with a tumor-like lesion below the stone. An endoscopic biopsy was performed during the operation, and the pathology report was chondrosarcoma. The tumor was heterologous and composed of epithelioid cell and chondrosarcoma components. The tumor’s immunoprofile was CK (+, only in the epithelioid tumor cells), vimentin (+, in both the epithelioid cells and chondrosarcoma cells), S100 (+, only in the chondrosarcoma cells), and GATA3 (−) (Figure 2). The picture was compatible with carcinosarcoma. Computed tomography (CT) of the abdomen revealed a tumor mass obstructing the upper ureter with proximal dilatation (Figure 3). 

She underwent laparoscopic nephroureterectomy and removal of the bladder cuff 1 month after the ureterorenoscopic surgery. The final pathology report was chondrosarcoma with invasion to periureteric fat. The tumor stage was pT3NX, cM0, stage III (AJCC, Eighth edition). She was then referred to our oncological department, where she received oral chemotherapy with tegafur 2# BID (tegafur 100 mg, uracil 224 mg, a 1:4 molar combination of ftorafur (tegafur) with uracil, which competitively inhibits the degradation of FU, resulting in sustained plasma and intratumoral concentrations). Due to poor renal function, other chemotherapy agents, such as doxorubicin and cisplatin, were not given. Follow-up CT in November 2022 revealed that there was no recurrence.

## 3. Discussion

Follow-up CT 7 months after surgery and chemotherapy revealed no recurrence in our patient, and therefore, the clinical outcome was fair after adequate treatment. However, as previously reported, the survival of chondrosarcoma varies from 2 month to over 10 years [8,9,10,11], a longer follow-up period is needed to assess her outcome as the initial stage was T3.

Chondrosarcoma is the second most common bone neoplasm (25%) following osteosarcoma (35%), and it is the most common malignant bone tumor in adults. Bone and joint cancer accounts for approximately 0.2% of all neoplasms in North America and Europe annually [12]. According to the histology, chondrosarcoma can be divided into conventional chondrosarcoma (85%), dedifferentiated chondrosarcoma (10%), mesenchymal chondrosarcoma (2%), clear cell chondrosarcoma (2%), and periosteal chondrosarcoma (1%) [13].

Mesenchymal chondrosarcoma is a rare variant of chondrosarcoma, accounting for only 2–10% of all chondrosarcomas. It is a highly aggressive tumor that has the potential to metastasize to the lungs and other sites, and it usually presents as a painless, slow-growing mass. While mesenchymal chondrosarcoma can affect individuals of any age, it typically presents in the second and third decades of life, with a median age of 22 years old. There is no significant difference in incidence between males and females [14,15]. The chondrosarcoma of the urinary system, on the other hand, is a relatively rare occurrence, accounting for less than 1% of all chondrosarcomas. It occurs equally in both sexes, but tends to present at a relatively older age than the chondrosarcoma of the bone, with a median age of 60 years old. However, it should be noted that patient demographics may differ from bone neoplasms, and further research is needed to better understand the epidemiology of urinary chondrosarcoma. When it comes to the treatment of mesenchymal chondrosarcoma, surgical resection is the mainstay of therapy, and adjuvant chemotherapy and/or radiation therapy may be considered for high-grade tumors or when complete surgical resection is not feasible. In the case of the chondrosarcoma of the urinary system, treatment typically involves surgical resection, often including a nephroureterectomy, and adjuvant chemotherapy may be considered for high-grade tumors or in cases where there is a risk of recurrence. Overall, while mesenchymal chondrosarcoma and chondrosarcoma of the urinary system may differ in terms of patient demographics and presentation, they are both aggressive tumors that require prompt diagnosis and aggressive management.

Mesenchymal chondrosarcoma mostly arises in the bone, and extra-skeletal mesenchymal chondrosarcoma was reported in about 39% of patients in a review of 111 cases [16]. Common extra-skeletal sites of invasion include the head and neck region, including the central nervous system, orbit, and nasal cavity, followed by the lower limbs and retroperitoneum. Primary mesenchymal chondrosarcoma in the kidneys and ureter is extremely rare, and few cases have been reported [5,8,9,10]. The first case of mesenchymal chondrosarcoma in the kidneys was reported in 1984 in a 27-year-old white male with gross hematuria and left flank pain radiating to the testis [6].

The symptoms of mesenchymal chondrosarcoma are usually pain and swelling, and may vary depending on the tumor size and location of the malignancy. Radiologically, calcification and osteolytic lesions can be observed in patients with intraosseous mesenchymal chondrosarcoma. Extra-skeletal lesions have no specific features, and may present as calcification with lobules [13,17]. The clinical presentation of our patient was a ureteral stone, and she had a past history of recurrent stone disease. In Taiwan, stone disease is highly associated with urothelial carcinoma [18].

Mesenchymal chondrosarcoma is a rare subtype of chondrosarcoma, and its histological features can be identified through gross and microscopic examinations, as well as immunohistochemistry. Grossly, mesenchymal chondrosarcoma tumors can be grey to pink with a firm texture. The tumors typically have a biphasic pattern with both small round or spindled undifferentiated cells and well-differentiated cartilaginous islands. The undifferentiated cells are usually arranged in a sheet-like pattern, while the cartilaginous islands are seen as well-circumscribed nodules. Microscopically, mesenchymal chondrosarcoma is characterized by a biphasic appearance of small round or spindled undifferentiated cells accompanied by well-differentiated cartilaginous islands. Immunohistochemistry is a useful tool in the diagnosis of mesenchymal chondrosarcoma. Positivity for vimentin in the undifferentiated regions indicates the mesenchymal origin of the tumor, while positivity for S-100 protein in the chondroid regions is the typical staining finding of mesenchymal chondrosarcoma [13]. In addition, the small round cell component of mesenchymal chondrosarcoma is positive for the cluster of differentiation (CD) 99 and Leu-7 (CD57, HNK-1), which morphologically and immunophenotypically mimic the Ewing sarcoma. However, the presence of hyaline cartilage and biomorphic features can help differentiate the mesenchymal chondrosarcoma from the Ewing sarcoma [19]. It is important to accurately diagnose the mesenchymal chondrosarcoma, as the tumor’s unique histological features and immunohistochemical characteristics affect the treatment and prognosis of the patient. Therefore, a thorough histological examination, coupled with appropriate immunohistochemical studies are essential for the diagnosis of mesenchymal chondrosarcoma.

The current treatment protocol for mesenchymal chondrosarcoma is based on the National Comprehensive Cancer Network (NCCN) guidelines, which recommend multi-agent chemotherapy in combination with local therapy for resectable tumors, followed by adjuvant chemotherapy and radiation therapy. The recommended chemotherapy regimen for mesenchymal chondrosarcoma is based on that for the Ewing sarcoma, with the use of vincristine, doxorubicin, and cyclophosphamide alternating with ifosfamide and etoposide. This combination has been shown to be effective in the treatment of the mesenchymal chondrosarcoma, and has been associated with improved survival rates. Surgery is the mainstay of treatment for the urinary chondrosarcoma, as seen in the case of the female patient with ureteral chondrosarcoma reported earlier. In cases of advanced disease, adjuvant radiation and chemotherapy may be necessary to achieve better outcomes [20,21]. However, the role of chemotherapy in the urinary chondrosarcoma treatment is not well defined, and its efficacy remains unclear due to the rarity of the disease. Gross and microscopic features of the mesenchymal chondrosarcoma, along with immunohistochemical stains, can help differentiate it from other small round cell tumors. The presence of hyaline cartilage and biomorphic features are characteristic of the mesenchymal chondrosarcoma and aid in distinguishing it from the Ewing sarcoma, another small round cell tumor that can have a similar immunophenotype. Thus, mesenchymal chondrosarcoma is a rare and aggressive tumor that requires a multimodal approach to treatment. Current treatment guidelines recommend multi-agent chemotherapy, local therapy, and adjuvant radiation therapy for resectable tumors. Surgery remains the primary treatment for urinary chondrosarcoma, while the role of chemotherapy in advanced cases is still unclear. It is important to differentiate the mesenchymal chondrosarcoma from other small round cell tumors using appropriate histopathological and immunohistochemical techniques to ensure appropriate treatment.

Cesari et al. compared the outcomes of 26 mesenchymal chondrosarcoma patients with a median age of 31 years, who underwent surgery with or without chemotherapy [22]. In their study, 24 patients underwent resection (n = 18) and amputation (n = 6). Twelve patients received chemotherapy, of whom nine had surgical complete remission, and the other 14 patients did not receive chemotherapy, of whom 12 had surgical complete remission. After 48 months of follow-up, the patients with surgical complete remission had a 27% overall survival rate in 10 years, while it was 0% in those who did not (*p* = 0.0007). In addition, the patients who received chemotherapy and had surgical complete remission had a 76% 10-year disease-free survival rate, compared to only 17% in the patients who did not receive chemotherapy. This study underscores the importance of chemotherapy after surgical resection.

Mesenchymal chondrosarcoma is a rare and aggressive neoplasm with a poor prognosis. According to a systematic review of 107 patients, the overall survival rates at 5, 10, 15, and 20 years were reported as 55.0%, 43.5%, 35.4%, and 15.7%, respectively. Moreover, the event-free survival rates were lower than 50% at 5 years of follow-up, and only 8.1% after 20 years [23]. Several studies have reported similar poor outcomes for patients with mesenchymal chondrosarcoma, with high rates of recurrence and metastasis even after radical surgery and adjuvant chemotherapy. Given the high malignancy and poor prognosis associated with the mesenchymal chondrosarcoma, a long-term follow-up is essential, even decades after the diagnosis. Close monitoring of patients is required, with regular imaging and surveillance for recurrence or metastasis. Additionally, multidisciplinary care, including close collaboration between urologists, pathologists, medical oncologists, and radiation oncologists, is crucial for the management of patients with mesenchymal chondrosarcoma. While the outcomes of the chondrosarcoma of the urinary system are generally poor, the prognosis may vary depending on various factors, such as tumor size, location, and stage, as well as the patient’s age, overall health, and response to treatment. Further cases are needed to clarify the prognosis and optimal management strategies for the chondrosarcoma of the urinary system [2]. Ongoing research and clinical trials are exploring new treatment approaches, such as targeted therapy and immunotherapy, which may offer hope for improved outcomes in the future.

In summary, the chondrosarcoma of the ureter is an exceptionally uncommon condition, and its clinical presentation can often make it challenging to differentiate from the more prevalent urothelial cell carcinoma. Additionally, the precise role of stone disease in the pathogenesis of this disease remains unclear. Despite these challenges, there is some promising evidence to suggest that oral chemotherapy with uracil may lead to a fair outcome for those suffering from this disease. However, it is important to note that additional observations of its clinical outcomes are necessary to gain a more comprehensive understanding of its effectiveness in treating the chondrosarcoma of the ureter.

## Data Availability

All of the data are available upon request to the corresponding author.
